# Varied Presentation of Congenital Segmental Dilatation of the Intestine in Neonates: Report of Three Cases

**DOI:** 10.21699/jns.v5i4.431

**Published:** 2016-10-10

**Authors:** Binod Kumar Rai, Bilal Mirza, Imran Hashim, Muhammad Saleem

**Affiliations:** 1 Department of Pediatric Surgery, The Children's Hospital and the Institute of Child Health, Lahore; 2 Department of Pediatric Surgery, Children Hospital Faisalabad

**Keywords:** Congenital segmental dilatation, Neonate, Pouch colon, Anorectal malformation, Pneumoperitoneum, Intestinal obstruction

## Abstract

Congenital segmental dilatation (CSD) of the intestine is a rare developmental anomaly characterized by sharply demarcated dilatation of a gastrointestinal segment and may present with intestinal obstruction. We report three cases of CSD of the intestine in neonates with varied presentation. First patient was mistaken as pneumoperitoneum on abdominal radiograph, which led to initial abdominal drain placement. The 2nd patient was a case of anorectal malformation associated with congenital pouch colon (CPC) and CSD of ileum; and the third case presented as neonatal intestinal obstruction and found to have CSD of ileum. All the patients were successfully managed in our department.

## INTRODUCTION

Congenital segmental dilatation of the intestine is a rare anatomical lesion characterized by three-to four-fold dilatation of a part of small or large intestine. There is an abrupt transition between normal afferent/efferent bowel loops and CSD.[1] Swenson and Rathauser first described three patients with segmental dilatation of the colon in 1959.[1] Ileum is affected mostly, followed by the colon, jejunum and duodenum.[2,3] We herein describe our experience of managing 3 cases of CSD of intestine in neonates.

## CASE SERIES

**Case 1:**

A 17–day-old, full term female neonate, weighing 2.6 kg, presented with abdominal distension and bilious vomiting for 3 days. She was passing stool. There was no delayed passage of meconium documented. On examination, she was sick looking, with tense, distended abdomen, and absent bowel sounds. On rectal stimulation, she passed small amount of stool. X-ray abdomen erect was interpreted as pneumoperitoneum (Fig. 1). All the laboratory parameters were within normal range. An intraperitoneal drain was passed; neither air nor stool was noted during drain insertion. At laparotomy, a dilated segment of colon starting from ileocecal junction to mid-transverse colon was found (Fig. 2). The involved segment (CSD) was resected and distal ileostomy with colonic mucous fistula was made. The postoperative recovery was uneventful and the child was discharged on 4th postoperative day. Histopathological examination of the specimen revealed colonic tissue with normal ganglion cells. 

**Figure:**
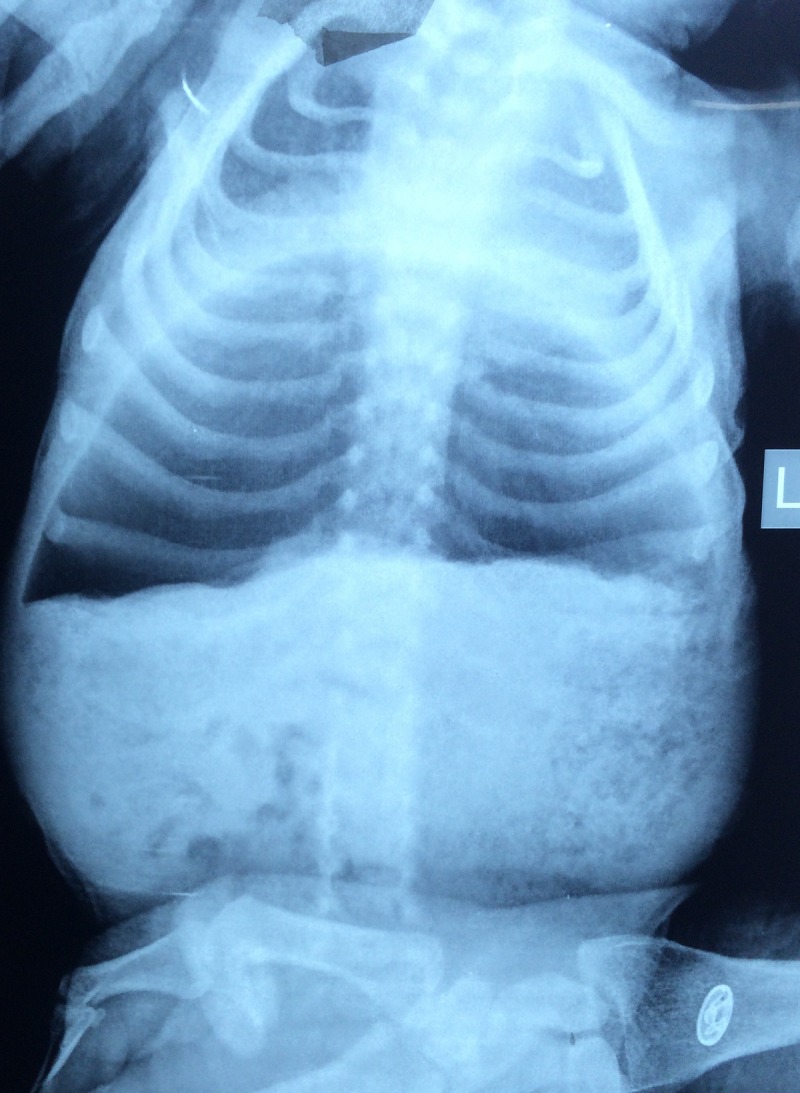
Figure 1: X-ray abdomen-erect showing huge air shadow in the upper abdomen.

**Figure:**
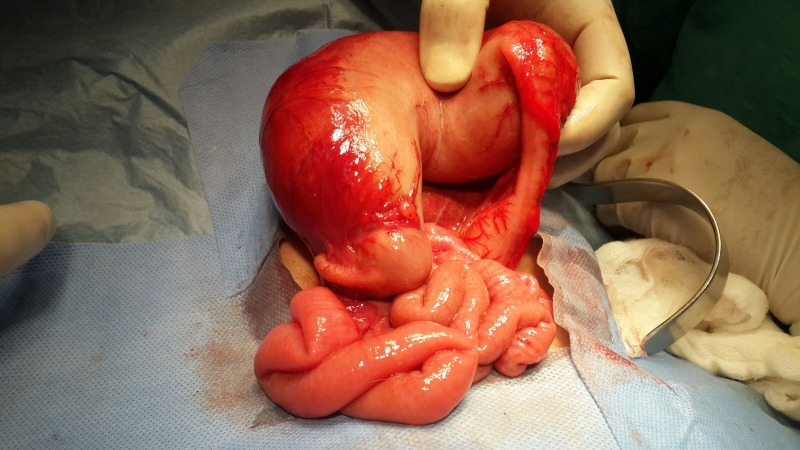
Figure 2: Segmental dilatation of the colon in the first case.

**Case 2:**

A 2-day–old, full term male neonate weighing 2.8 kg, presented with absent anal opening for which a colostomy was made. On 2nd postoperative day, the colostomy was found to be sunken. At re-exploration, a type IV pouch colon with segmental dilatation of the mid ileum (Fig. 3) was found. The previous colostomy was found to be exteriorized wall of the CPC (window colostomy). The pouch colon and dilated ileal segment were resected. An end-to-end ileal anastomosis and end colostomy was performed. The baby recovered well. Biopsy of the resected ileum and pouch colon showed presence of ganglion cells. The patient is waiting for definitive surgery. 

**Figure:**
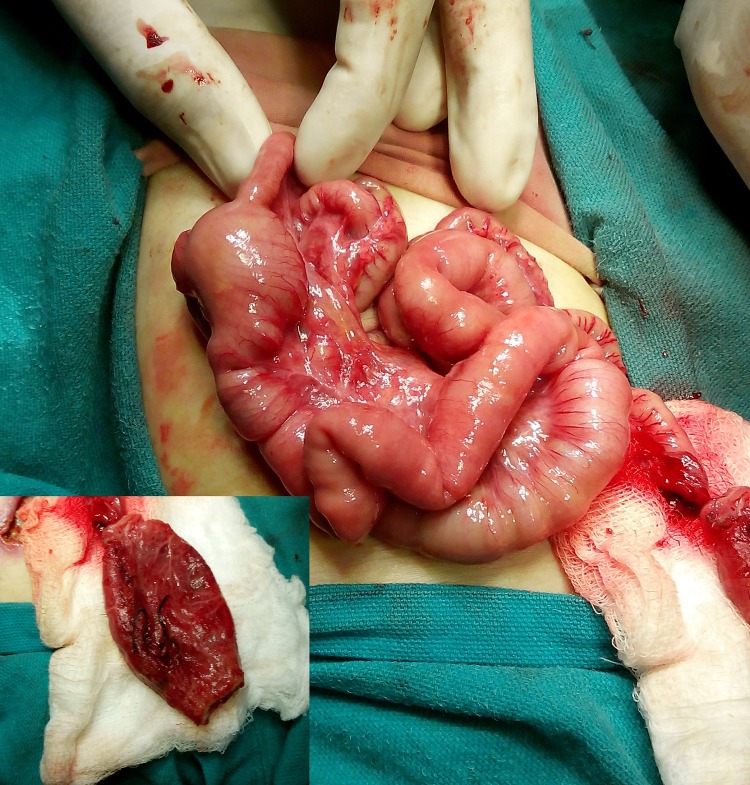
Figure 3: Segmental dilatation of ileum. Inset shows resected CPC.

**Case 3:**

A 3-day-old, full term male neonate weighing 2.4 kg, presented with bilious vomiting, abdominal distension and failure to pass meconium since birth. Examination revealed mild abdominal distension with empty rectum. Erect x-ray abdomen showed dilated bowel shadows. With provisional diagnosis of small bowel atresia, the child was operated upon. It revealed a segmental dilatation of the terminal ileum which was resected and end-to-end ileo-ileal anastomosis was done (Fig. 4). The postoperative recovery was uneventful. Biopsy of the resected ileum showed presence of ganglion cells. 

**Figure:**
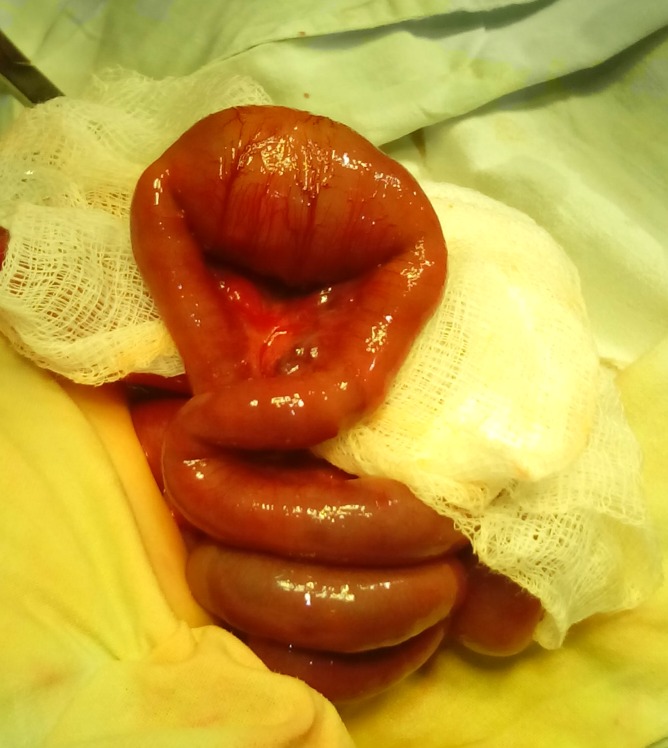
Figure 4: Segmental dilatation of the ileum in the third case.

## DISCUSSION

Neonates with CSD present mostly with intestinal obstruction whereas in older children the presentation is with abdominal pain, constipation, anemia, and failure to thrive.[4] CSD of intestine has been reported in association with a number of other anomalies such as anorectal malformation (including rectal atresia and congenital pouch colon), intestinal atresia, duplication cyst, cleft lip and palate etc.[3,5,6] In our first patient, the patient had abdominal distension and abdominal radiograph was misinterpreted as pneumoperitoneum. Our second case was associated with anorectal malformation and type IV CPC. The third case was provisionally diagnosed as intestinal atresia. Thus, in all of our cases, the CSD of intestine was an operative finding as supported by the literature where majority of neonatal cases are diagnosed peroperatively.[5] All the reported cases have mentioned a single segmental dilatation, however a case of multiple segmental dilatation of colon associated with cleft lip and palate, and rectal atresia has also been reported in literature.[6]

CSD of intestine can be detected as an intra-abdominal cystic mass on prenatal ultrasonography.[7] Abdominal x-ray may reveal a huge dilated bowel shadow with or without air-fluid level and can easily be misdiagnosed as other surgical entities as happened in our first case.[4] Contrast enema studies may be performed to differentiate it from other gastrointestinal anomalies.

CSD of colon in association with CPC in a patient of anorectal malformation is termed as type V CPC. Chadha et al [8] endorsed to consider CPC associated with CSD of intestine, rectal atresia, prune belly syndrome, or pseudoexstrophy as type V CPC. In our 2nd case, CPC was associated with a CSD of ileum which is not reported hitherto. We also second Chadha et al endorsement and suggest to consider this concurrence (CSD of ileum and CPC) under the category of type V CPC. 

The treatment of congenital segmental dilatation of intestine is resection of the dilated segment and end-to-end anastomosis.[4] Temporary stomas can be made in critically sick patients. Histology of the resected segment is usually normal in most of the cases, however, some may show hypertrophied or very thin muscle layer in the involved segment.[4]

In conclusion, segmental dilatation of intestine is a rare entity and can have variable presentation in neonates. It has strong preponderance to other associated anomalies. Outcome is promising when dealt surgically with resection of the involved bowel.

## Footnotes

**Source of Support:** Nil

**Conflict of Interest:** None
